# Thiopurines: Recent Topics and Their Role in the Treatment of Inflammatory Bowel Diseases

**DOI:** 10.3389/fphar.2020.582291

**Published:** 2021-01-29

**Authors:** Keiichi Tominaga, Takeshi Sugaya, Takanao Tanaka, Mimari Kanazawa, Makoto Iijima, Atsushi Irisawa

**Affiliations:** Department of Gastroenterology, Dokkyo Medical University, Tochigi, Japan

**Keywords:** inflammatory bowel disease, ulcerative colitis, crohn's disease, thiopurine, pharmacological action, biological agents

## Abstract

Ulcerative colitis (UC) and Crohn's disease (CD) are chronic inflammatory bowel diseases (IBD) of unknown etiology, characterized by repeated relapse and remission. The efficacy of thiopurine in IBD was first reported in the late 1960s. Thiopurines are used to alleviate the symptoms of IBD, especially UC. These drugs have a steroid-sparing potential and are widely used for the purpose of maintaining long-term remission in steroid-dependent cases. Therefore, thiopurines tend to be used long-term, but adverse events that accompany long-term use, such as lymphoproliferative disorders, must be monitored with care. In contrast, thiopurine plays a critical role in controlling the immunogenicity of biologics. Furthermore, although thiopurine is an old drug, new findings, including the prediction of serious adverse events such as severe alopecia and acute advanced leukopenia, by nudix hydrolase 15 gene polymorphism analysis, as well as the possibility of appropriate drug monitoring by detailed analysis of 6-thioguanine nucleotides have been clarified. However, the consequences of thiopurine withdrawal have not been determined and further studies, including randomized controlled trials, are necessary to answer the clinical question regarding the scenarios in which thiopurine withdrawal is possible.

## Introduction

Ulcerative colitis (UC) and Crohn’s disease (CD) are chronic inflammatory bowel diseases (IBDs) with unknown etiology, characterized by repeated relapse and remission (Ghouri et al., 2020). Rapid induction and ensured maintenance of remission are the foundations of IBD treatment. In UC, remission maintenance therapy is most commonly based on 5-aminosalicylic acid (5-ASA) monotherapy. Contrarily, in cases where it is difficult to maintain remission with 5-ASA monotherapy, immunomodulators (IMs) are used to attain long-term steroid-free remission after initial induction of remission with steroids (Takahashi et al., 2016; Timmer et al., 2016; Matsuoka et al., 2018). IMs are also important for the treatment of CD, where they are used to maintain long-term steroid-free remission and prevent postoperative recurrence (Takahashi et al., 2016; Timmer et al., 2016; Kakuta et al., 2018a; Matsuoka et al., 2018; Gjuladin-Hellon et al., 2019).

The effectiveness of IMs in treating IBD was first reported in the late 1960s (Brooke et al., 1969). Since then, the efficacies of IMs such as azathioprine (AZA), 6-mercaptopurine (6-MP), methotrexate, and mycophenolate mofetil have been reported (Smith and Cooper, 2014; Wang et al., 2015). The use of AZA and 6-MP for UC and CD is recommended by the Japanese clinical guidelines (Matsuoka et al., 2018). Herein, we describe the pharmacological actions of thiopurines and their interactions with other drugs. Moreover, we discuss the positioning of thiopurines in the treatment of IBD, as well as the benefits and risks of thiopurine treatment.

### Pharmacological Action and Interaction of Thiopurines

**FIGURE 1:**
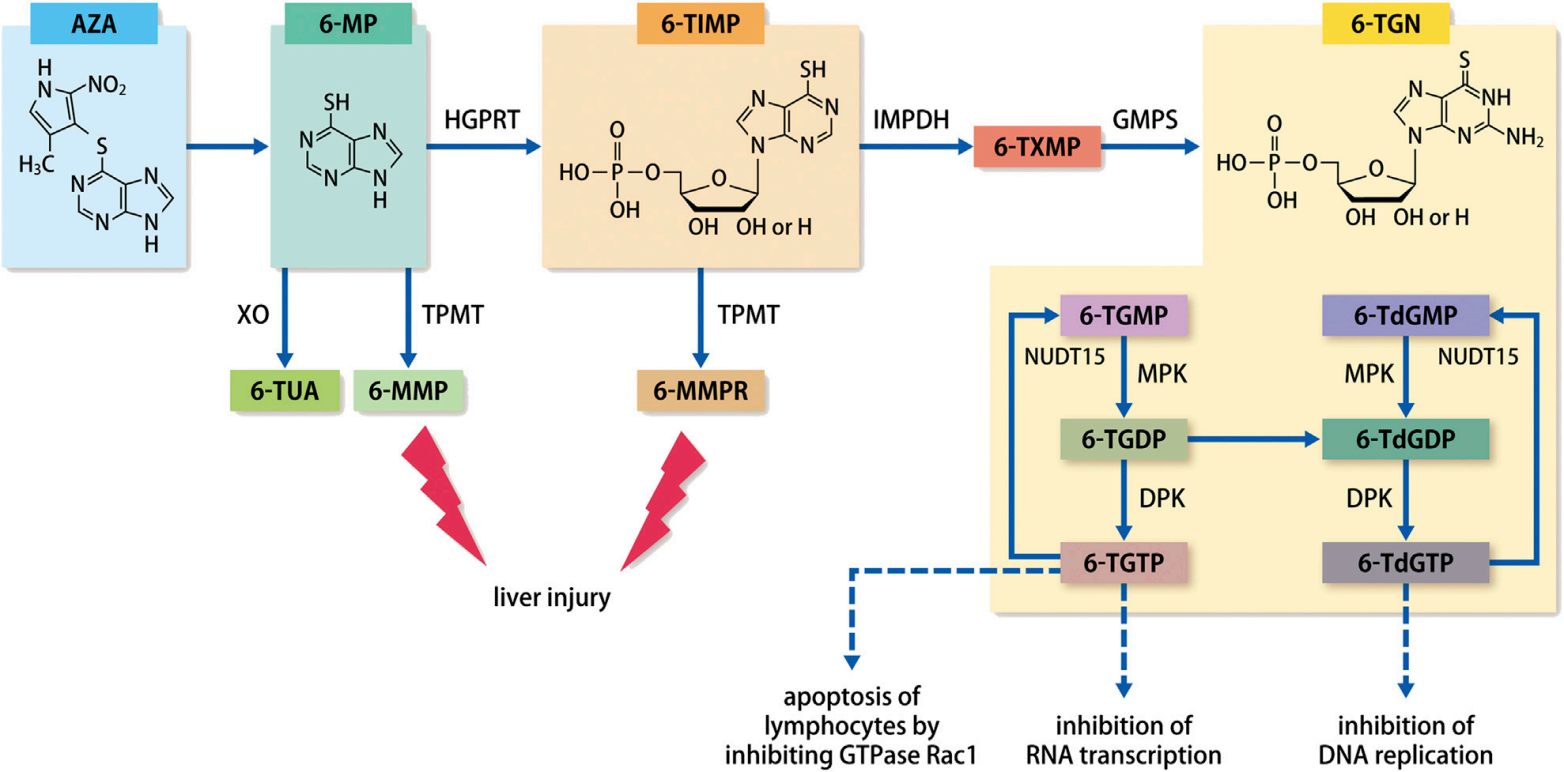
Metabolic pathways of thiopurines. Abbreviation: AZA, azathioprine; 6-MP, 6-mercaptopurine; 6-MMP, 6-methylmercaptopurine; 6-TUA, 6-thiouric acid; 6-TIMP, 6-thioinosine monophosphate; 6-MMPR, 6-methylmercaptopurine ribonucleotides; 6-TXMP, 6-thioxanthosine monophosphate; 6-TGN, 6-thioguanine nucleotides; 6-TGMP, 6-thioguanosine monophosphate; 6-TGDP, 6-thioguanosine diphosphate; 6-TGTP, 6-thioguanosine triphosphate; 6-TdGMP, 6-thio-deoxyguanosine monophosphate; 6-TdGDP, 6-thio-deoxyguanosine diphosphate; 6-TdGTP, 6-thio-deoxyguanosine triphosphate; TPMT, thiopurine S-methyltransferase; XO, xanthine oxidase; HGPRT, hypoxanthine guanosine phosphoribosyl transferase; IMPDH, inosine monophosphate dehydrogenase; GMPS, guanosine-monophosphate synthetase; MPK, monophosphate kinase; DPL, diphosphate kinase; NUDT15, Nudix hydrolase 15.

The metabolic pathways of thiopurines are illustrated in Figure 1. AZA is a prodrug that after absorption into the body via the gastrointestinal tract, is promptly converted nonenzymatically to 6-MP which is subsequently metabolically regulated by xanthine oxidase (XO), thiopurine S-methyltransferase (TPMT), and hypoxanthine guanosine phosphoribosyl transferase (HGPRT) (Derijks et al., 2006). Various studies have reported that the combination of XO inhibitors, allopurinol and thiopurines, increased blood concentrations of 6-thioguanine nucleotides (6-TGN) following its conversion from 6-thioinosine monophosphate (6-TIMP), thereby reducing the dose of thiopurines and enhancing the immunosuppressive effects (Sparrow et al., 2005; Sparrow et al., 2007; Pavlidis et al., 2016). Moreover, 6-MP is converted to 6-methylmercaptopurine (6-MMP) by TPMT; 6-MMP is reportedly associated with an increased risk of liver injury in red blood cells (RBCs) at levels ≥5700 pmol/8 ×10^8^ RBC (Wang and Weinshilboum, 2006; Colleoni et al., 2013). The TPMT gene exhibits a multitude of genetic polymorphisms that affect its enzymatic activity. In particular, more than 30 TPMT gene variants that affect its activity have been reported (Wang and Weinshilboum, 2006; Colleoni et al., 2013). Numerous studies have been performed to address the clinical issues arising from leukopenia in patients with low TPMT activity, as the condition develops due to elevated levels of 6-TGN (Roblin et al., 2011). Polymorphisms in TPMT predominantly result from variant alleles that arise from single nucleotide polymorphisms. Compared with the wild-type TPMT, the TPMT variants are significantly unstable and have lower activity. Based on the assessment of the genotype and TPMT activity, guidelines have been formulated in the US and Europe for determining the initial doses of thiopurines (Relling et al., 2019). However, the activity of TPMT varies with ethnicity. While the standard doses of AZA and 6-MP in the US and Europe are 1.5–2.5 mg/kg and 1.0–1.5 mg/kg, respectively, the doses in Japan are 1.0 mg/kg and 0.6 mg/kg, respectively. This is due to the fact that a study confirmed that Asian patients commonly have low TPMT activity (Gomollón et al., 2017; Matsuoka et al., 2018). However, another study reported that the levels of 6-TGN in blood are maintained at a therapeutic level in the majority of Japanese IBD patients (Komiyama et al., 2008).

As an active metabolite of thiopurines, 6-TGN ultimately exhibits its pharmacological effects in the form of 6-thio-(deoxy)guanosine-triphosphate (6-T(d)GTP). Incorporation of 6-T(d)GTP, which is structurally similar to guanine (a nucleic acid-composing base), into DNA or RNA during chromosome replication or gene transcription leads to the exhibition of its primary role in blocking nucleic acid synthesis and activating lymphocyte proliferation. Previously, blood concentrations of 6-TGN were thought to correlate with the effectiveness of IBD treatment, with numerous studies reporting on the utility of monitoring 6-TGN concentrations (Cuffari et al., 1996; Dubinsky et al., 2000; Osterman et al., 2006; Komiyama et al., 2008). Dubinsky reported that a high concentration of ≥450 pmol/8 ×10^8^ RBC is linked to a high risk of bone marrow suppression (Dubinsky, 2004). A meta-analysis by Osterman showed that ≥230–260 pmol/8 ×10^8^ RBCs correlated substantially with clinical remission (Osterman et al., 2006). Contrarily, 6-TGN concentrations and effectiveness did not correlate strongly, with large variations potentially causing issues, and suggested that non-6-TGN metabolites/intermediates with immunomodulatory effects may be involved (Lowry et al., 2001; Goldenberg et al., 2004).

### *NUDT15* Polymorphism

Questions relating to the 6-TGN concentration and pharmacological effects of thiopurines were later clarified in studies evaluating nudix hydrolase 15 (*NUDT15*) gene polymorphisms. After thiopurine treatment, Asian patients occasionally present with acute advanced leukopenia combined with advanced alopecia as an adverse event (AE), which is rarely reported in Western populations (Gearry et al., 2004; Palmieri et al., 2007). For this reason, genetic background has been thought to influence Asian-specific thiopurine-related side effects. With recent advances in genome analysis technology, conducting genome-wide correlation analysis has become feasible with relative ease. In 2014, Yang et al. reported that in Korean patients with CD, advanced leukopenia showed an extremely strong correlation with *NUDT15* polymorphism (Yang et al., 2014). Several studies on Asian populations soon followed in the fields of blood disorders, neurological disorders, skin disease, and IBD (Asada et al., 2016; Moriyama et al., 2016; Tanaka et al., 2015; Lee et al., 2016; Liang et al., 2016; Zhu et al., 2016; Kim et al., 2017; Shah et al., 2017; Soler et al., 2018; Kishibe et al., 2018. However, all cases were related to *NUDT15* codon 139 gene polymorphism, and almost all patients with homozygous risk (Cys/Cys type) presented with severe leukopenia and complete alopecia. An IBD study on Japanese patients revealed that complete alopecia was not only associated with severe leukopenia but was also strongly associated with a *NUDT15* polymorphism (Kakuta et al., 2016). *NUDT15* polymorphisms occur at a very low frequency in Europeans and Africans (Moriyama et al., 2016). The allele frequency of another *NUDT15* variant, *p.Gly17_Val18del*, is approximately 2% in Europeans; this variant is also reported to correlate with thiopurine-induced leukopenia (Walker et al., 2019). Nevertheless, none of these reports fully describe the association between complete alopecia and *NUDT15* polymorphisms. Given that alopecia is an extremely rare AE in Caucasians patients taking thiopurines, complete alopecia may be considered a problem specific to Asian populations. In particular, complete alopecia may pose a major problem for young patients, as it can manifest not only as scalp hair loss, but also as entire body hair loss, including eyebrows and armpit hair.

Recently, Kakuta et al. reported a large-scale observational study (MENDEL study) on AEs following the treatment of IBD with thiopurines in Japanese patients (Kakuta et al., 2018b). This Japanese multicenter study analyzed 1,291 Japanese patients with IBD with a history of taking thiopurines and showed that *NUDT15* codon 139 gene polymorphism correlated with severe leukopenia and alopecia. This observation led to the inclusion of *NUDT15* codon 139 polymorphism detection assays under Japanese national health insurance coverage for thiopurine-naïve patients. Thiopurines are not only used to treat IBD but also many autoimmune diseases, and during immunosuppressive therapy after organ transplantation, the ability to predict whether thiopurines will induce early leukopenia and severe alopecia is welcome news for patients. When treating patients with IBD, it is also recommended that thiopurines are avoided in known carriers of the homozygous risk allele (Cys/Cys) at the *NUDT15* codon 139 gene. In heterozygous risk allele (Arg/Cys) carriers, thiopurines are started at approximately half the normal dose, whereas in patients with the wild-type allele (Arg/Arg) thiopurines are started at the normal dose (Kakuta et al., 2020).

Patients with *NUDT15* polymorphism may exhibit leukopenia as a dose-dependent AE. Therefore, the concentration of thiopurine metabolite 6-TGN was initially predicted to be elevated. However, Asada et al. reported that 6-TGN levels did not fluctuate in patients with *NUDT15* polymorphisms (Asada et al., 2016). A study of pediatric cases of acute lymphoblastic leukemia showed that NUDT15 is an enzyme that dephosphorylates the final active metabolite of thiopurines, 6-T(d)GT, to 6-thio-(deoxy) guanosine-monophosphate (6-T(d)GMP). Moreover, the report showed that coding polymorphisms in *NUDT15* suppressed this dephosphorylating function and prevented 6-TGTP and 6-TdGTP from being converted to 6-TGMP and 6-TdGMP. This led thiopurine to exhibit excessive pharmacological effects that were manifested principally as lymphocyte apoptosis via Rac1 inhibition and inhibition of RNA transcription and DNA replication, ultimately causing severe leukopenia (Moriyama et al., 2016). Measurement of 6-TGN concentration includes both 6-T(d)GMP and 6-T(d)GTP; levels of 6-TGN*,* which had previously been used as an index, do not change with *NUDT15* polymorphism. 6-TGTP is incorporated into RNA, and 6-T(d)GTP is incorporated into DNA, where it inhibits RNA transcription and DNA replication, respectively, thereby causing cell apoptosis. 6-TGTP also causes T-lymphocyte apoptosis by inhibiting the activation of GTPase Rac1 (Tiede et al., 2003; Marinković et al., 2014). Therefore, it is suggested that measurement of 6-T(d)GTP concentration is appropriate for efficient drug blood monitoring.

### Effects of Thiopurines in Maintaining Remission

Prospective data showing the effectiveness of thiopurine monotherapy in inducing remission in UC remains limited. Ardizzone et al. conducted a single-center blinded control study of 5-ASA at 3.2 g/day group or AZA at 2 mg/kg/day group, given the intention of inducing remission in steroid-dependent patients with UC. They observed that the remission maintenance rate was 19.4% in the 5-ASA group and 52.6% in the AZA group, demonstrating that AZA had a substantially greater remission rate as well as positive effects in reducing steroids (Ardizzone et al., 2006). However, a considerable amount of time is required for thiopurines to exert their effects (Present et al., 1980), and in actual clinical practice, this agent is infrequently used to induce remission and is more often used in conjunction with steroid reduction in steroid-dependent cases or to maintain remission.

Accumulating evidence from randomized controlled trials (RCTs) and meta-analyses have shown the effects of thiopurines in maintaining remission (Hawthorne et al., 1992; Hibi et al., 2003; Gisbert et al., 2009; Timmer et al., 2016). A comparison between AZA and placebo in the Cochrane Database Systematic Review indicated an odds ratio for remission maintenance failure of 0.41 (95% confidence interval, 0.24–0.70) (Timmer et al., 2016). A meta-analysis by Gisbert et al. showed that remission maintenance was achieved in 60% of patients with UC taking thiopurines, but in only 37% taking the placebo or 5-ASA, demonstrating the benefits of thiopurines (Gisbert et al., 2009).

The efficacy of thiopurines in maintaining remission in patients with CD has also long been demonstrated. Candy et al. investigated AZA in conjunction with steroid therapy in patients with CD and reported a markedly higher proportion of patients in remission in the AZA group than in the placebo group after 15 months (Candy et al., 1995).

Recently, powerful immunosuppressive therapies have been introduced to maintain remission, such as anti-tumor necrosis factor-alpha (anti-TNF-α) antibodies and Janus kinase (JAK) inhibitors. These therapies are used in moderate to severe cases with high disease activity as they can induce and subsequently maintain remission. However, in steroid-dependent cases, many guidelines and position statements recommend that an attempt be made to maintain remission using thiopurines prior to these biological agents and JAK inhibitors due to concerns over infection risks and malignancy since many of these expensive drugs are administered via injection (Harbord et al., 2017; Matsuoka et al., 2018; Lamb et al., 2019).

### Relationship of Thiopurines With Biological Agents and Small-Molecule Drugs

By virtue of the introduction of biological agents, clinical treatment for IBD has made considerable progress. At the current height of biological agents, thiopurines are attracting attention for their pharmacological effects for the loss of response (LOR) (Gisbert and Panés, 2009; Bartelds et al., 2011; van Schouwenburg et al., 2013).

Infliximab (IFX) was the first biological agent introduced to treat IBD; however, as it showed characteristics of a chimeric biological agent due to the approximately 25% mouse genes, whether this agent should be started as monotherapy or in combination with thiopurines remains contentious. In the SONIC study, the effects of thiopurine monotherapy, IFX monotherapy, and combination therapy of IFX and thiopurines were compared in patients with CD who had not received biological agents or thiopurines (Colombel et al., 2010). This study demonstrated that the clinical remission rate at 26 weeks was substantially greater in the combination therapy group than in the monotherapy group. The appearance ratio of anti-biological antibodies was low in the combination therapy group, and decreased blood concentration of biological agents were confirmed to markedly affect treatment outcomes. The “SUCCESS” RCT, that was similar to the SONIC study, was conducted on 239 patients with moderate to severe UC, who did not receive biologics or thiopurine drugs. The main outcome of that study was the steroid-free remission rate at 16 weeks after starting treatment in the IFX monotherapy, thiopurine monotherapy, and combination therapy groups. The remission rates were 24%, 22%, and 40%, respectively, demonstrating that the steroid-free remission rate was markedly greater in the combination therapy group (Panaccione et al., 2014). These results, similar to the aforementioned SONIC study, indicated not only the substantial involvement of thiopurines suppressing the immunogenicity of IFX, but also some effects of thiopurine itself. A meta-analysis that included four articles indicated that the SUCCESS trial, post-hoc analysis of ACT1 and 2, suggested that regardless of previous use of thiopurines, the combination therapy showed a substantially greater remission rate at 4–6 months, although the remission rate at 12 months was not starkly different between combination therapy and monotherapy (Lichtenstein et al., 2009; Christophorou et al., 2015). Presently, the principal biological agents used for treating IBD are human-type biologics and biosimilars. The DIAMOND study is a Japanese RCT which examined the efficacy of thiopurines in combination with a human-type biologic (Matsumoto et al., 2016). This study investigated the efficacy of adalimumab (ADA), a human-type anti-TNF-α antibody preparation, in combination with thiopurine, for patients with moderate to severe CD who were naïve to both biologics and thiopurines. However, the DIAMOND study did not elucidate the differences between combination therapy and monotherapy. Although no statistically significant differences were observed in the ADA trough levels and anti-ADA antibody levels, the combination group which was administered ADA and thiopurine, exhibited higher ADA trough levels, and contained a lower proportion of patients positive for anti-ADA antibodies compared to the monotherapy group.

Furthermore, as a different concept from SONIC, SUCCESS, and DIAMOND study, some study has been made of whether the additional administration of thiopurine restores therapeutic responsiveness when LOR occurs with anti-TNF-α antibody monotherapy. Macaluso et al. reported an analysis of 46 IBD patients treated with anti-TNF-α antibody and additionally treated with thiopurines, methotrexate, and mycophenolate mofetil (Macaluso et al., 2018). Results of this study show that additional combination therapy was effective in 42.4 of CD and 53.8% of UC. Moreover, the average dose of AZA used at that time was a low dose. These results indicate that the addition of low-dose IM is an effective and safe optimization strategy when LOR occurs with anti-TNF-α antibody monotherapy.

Small-molecule drugs are believed to not elicit anti-drug antibodies, and hence do not induce a LOR via anti-drug antibodies. The JAK inhibitor, tofacitinib, is a small-molecule drug. A report indicated that a stronger therapeutic effect was achieved following the administration of thiopurines in combination with tofacitinib. However, the design of the large-scale OCTAVE trial did not allow tofacitinib to be used in combination with thiopurines; hence, the safety of two-agent therapy has yet to be verified. Additionally, the use of thiopurines in combination with tofacitinib has not been recommended due to the risk of infection, particularly viral infection, associated with tofacitinib itself, and concerns that two-agent therapy may induce excessive immunosuppression that could break down the intestinal barrier, thereby making it difficult to control disease activity (Sandborn et al., 2017; Xiong et al., 2019; Feng et al., 2020). Clear evidence is currently lacking on the clinical significance of long-term combination use of biological agents and thiopurines.

### Thiopurine-Associated AEs Are Not Linked to *NUDT15* Polymorphisms

Various AEs are often observed with thiopurine treatment. The frequency of AEs in patients with IBD varies from 9% to 35%, depending on the study (Fraser et al., 2002; Timmer et al., 2016; Kakuta et al., 2018; Gjuladin-Hellon et al., 2019). Table 1 shows the frequency of AEs after thiopurine treatment in IBD (Kakuta et al., 2018; Pugliese et al., 2018; Özgenç et al. 2018; Calafat et al., 2019). Moreover, the appearance of AEs is substantially related to racial differences (Kakuta et al., 2018). AEs are generally categorized into two types: dose-dependent AEs that occur from the pharmacological actions of AZA/6-MP, and dose-independent AEs that are responses to the drug itself. Decreased blood cells, mild alopecia, increased infection susceptibility, and hepatic dysfunction are considered to be dose-dependent AEs, whereas fever, drug eruptions, joint pain, gastrointestinal symptoms, malaise, and acute pancreatitis are considered dose-independent AEs. However, the classification of many AEs is unclear. Gastrointestinal AEs such as nausea, vomiting, and decreased appetite are commonly caused by the compositional binding of the imidazole ring of AZA, and a switch to 6-MP can be beneficial (Kennedy et al., 2013; Calafat et al., 2020). Although conditions can alleviate with time, or proton pump inhibitors and gastrointestinal motility-improving drugs can be clinically effective, thiopurines must be discontinued if symptoms do not improve even after such treatment.

**TABLE 1:** Adverse event rates for thiopurines in patients with IBD.

Ref.	Disease	Case	Leukopenia	Alopecia	Hepatic toxicity	Pancreatic toxicity	Digestive intolerance	Infection	Malignancy
Kakuta et al. (2018)	IBD	1,282	18.2% (*n* = 233)	6.8% (*n* = 87)	3.7% (*n* = 47)	1.7% (*n* = 20)	7.2% (*n* = 92)	1.3% (*n* = 17)	0.2% (*n* = 2)
Calafat et al. (2019)	IBD	17,365	6.5% (*n* = 1,134)		5.1% (*n* = 890)	4.2% (*n* = 722)	10.3% (*n* = 1782)		
Pugliese et al., (2018)	UC	45	28.9% (*n* = 13)	2.2% (*n* = 1)	13.3% (*n* = 6)	6.7% (*n* = 3)	28.9% (*n* = 13)	37.8% (*n* = 17)	4.4% (*n* = 2)
Özgenç et al. (2018)	UC	48	2.1% (*n* = 1)				6.3% (*n* = 3)		

IBD, inflammatory bowel disease; Ref., reference; UC, ulcerative colitis.

### Involvement of Long-Term Thiopurines and Onset of Lymphoproliferative Disorders (LPDs)

Thiopurines are commonly used to maintain remission in clinical care for patients with IBD. Consequently, the medication period is inevitably prolonged. Concerns have been raised regarding the increased risk of LPDs such as malignant lymphoma after long-term thiopurine use. According to the CESAMI study, the incidence of LPDs in patients with IBD with no history of taking thiopurines and patients with IBD taking thiopurines were 0.26/1,000 patients/year and 0.90/1,000 patients/year, respectively, indicating an increased risk of thiopurines (Beaugerie et al., 2009). Table 2 presents a summary of several articles describing studies assessing the risks of developing lymphoproliferative disorders when using thiopurines in IBD patients (Beaugerie et al., 2009; Ashworth et al., 2012; Khan et al., 2013; Pasternak et al., 2013; Beigel et al., 2014; Fukata et al., 2014; Wang et al., 2016).

**TABLE 2:** Occurrence of lymphoproliferative disorders attributable to thiopurines in patients with IBD.

Ref [Ref. No]	Case	TP use	Previous TP use	TP never use	TP use (%)	Disease
Beaugerie et al. (2009)	23/19,486	6/5,867	2/2,809	15/10,810		UC, CD, and unclassified IBD
Ashworth et al. (2012)	2/1,374	2/839		0/535		UC, CD, and unclassified IBD
Pasternak et al. (2013)	95/43,969	9/5,197				UC and CD
Khan et al. (2013)	142/45,693	18/4,734	5/4,662	119/36,297		UC
Beigel et al. (2014)	4/666	3/262	1/374	0/30		UC and CD
Fukata et al. (2014)	12/10,500	2/1,341		10/9,159		UC
Wang et al. (2016)	10/2,663				8.7	UC

IBD, inflammatory bowel disease; Ref, reference; TP, thiopurines; UC, ulcerative colitis; CD, Crohn’s disease.

Non-Hodgkin’s lymphoma (NHL) comprises the majority of cases of lymphoma in patients with IBD. Moreover, most of these cases test positive for Epstein-Barr virus (EBV). A recent systematic review reported that NHL was the most common histological type (83.9%) in the IBD patients. Furthermore, EBV positive status was observed in a large part of lymphoma patients with IBD (44–75%) (Muller et al., 2020). Patients with IBD who receive thiopurines are likely to develop a subset of lymphoma similar to EBV-related malignant lymphoma that develops after organ transplantation. The prognosis of this subset of lymphoma is poor. Every precaution must be taken for patients who test negative for EBV, especially young male patients, as they are at high risk of developing the aforesaid lymphoma (Kotlyar et al., 2015; Beaugerie et al., 2020). Given the risk of LPDs in patients with IBD who receive thiopurines, it is important to examine them for EBV infection, regardless of whether the results are positive or negative.

Long-term use of thiopurines represents a substantial risk factor for the onset of LPD, but this risk is thought to decrease when thiopurines are discontinued, depending on the primary disease condition. However, UC relapse is obviously a concern with such discontinuation or withdrawal. According to Moreno-Rincón et al., in a retrospective cohort study examining a median sustained remission period of 33 months in patients with UC not on steroid treatment, relapse was observed after thiopurine withdrawal in 32.4% of patients, and the cumulative relapse rate was 18.9% at 12 months, 36.5% at 36 months, and 43.0% at 60 months (Moreno-Rincón et al., 2015). Similarly, Cassinotti et al. conducted a long-term survey in 155 patients with UC who discontinued AZA during the remission period and found relapse in one-third of patients after 1 year and in half of the patients after 2 years (Cassinotti et al., 2009). We have also previously demonstrated that the relapse rate is greater in the withdrawal group when comparing remission maintenance rates between thiopurine continuation and withdrawal groups of patients with UC with long-term sustained remission, and showed that endoscopic remission (Mayo endoscopic subscore 0) did not guarantee the absence of relapse after withdrawal (Takenaka et al., 2019). As described, a high relapse risk clearly exists with the withdrawal of thiopurines administered as remission maintenance therapy. No clear conclusion has yet been reached on the appropriate and optimal timing of withdrawal, especially considering the concerns for various complications including LPDs after long-term treatment. Nonetheless, due to the various AEs described above, it is necessary to consider thiopurine withdrawal in accordance with disease conditions. Furthermore, thiopurine withdrawal needs to be justified based on assessment of remission with new imaging-enhanced endoscopy or molecular biological examination of tissue samples. Kjærgaard et al. used mucosal biopsy specimens from patients with quiescent UC to analyze mucosal healing in quiescent UC at the molecular and functional level, and reported upregulations of tight junction proteins *claudin-2* and *claudin-4* mRNA levels, and downregulation of cyclooxygenase-1 enzyme mRNA levels (Kjærgaard et al., 2020). The use of tissue samples to explore mucosal molecular signatures may aid the assessment of remission and could potentially help in deciding when to temporarily withhold thiopurine treatment.

### Use of Thiopurines in IBD Patients During the Coronavirus Disease 2019 (COVID-19) Pandemic

A case of severe acute respiratory syndrome coronavirus 2 (SARS-CoV-2) infection was first reported in December 2019 (Zhu et al., 2020). SARS-CoV-2 is the causative agent of the global COVID-19 pandemic in 2020. Older adults; people with underlying diseases, including diabetes and cardiovascular disease; and patients with cancer are at a high risk of COVID-19. These patients are likely to develop severe symptoms and have a high mortality rate (Liang et al., 2020; Xie et al., 2020; Tian et al., 2021). Therefore, there is an increasing concern among health care professionals regarding the effect of immunosuppressive therapy on patients with IBD who receive corticosteroids, thiopurines, or biological agents. However, there is no evidence to indicate that the suffering from IBD increases the risk of contracting COVID-19. Practice guidelines from organizations such as the International Organization of IBD, the European Crohn’s and Colitis Organization, the British Society of Gastroenterology, and the American Gastroenterological Association recommend that treatment be continued for IBD patients in remission, even during the COVID-19 pandemic, so that remission can be maintained (Dotan et al., 2020; Kennedy et al., 2020; Rubin et al., 2020). These guidelines state that the discontinuation of immunosuppressive therapy for such patients for the purpose of preventing SARS-CoV-2 infections may lead to the use of higher doses of corticosteroids in the case of relapse of IBD, which may expose the patients to a higher risk than that otherwise (Dotan et al., 2020; Kennedy et al., 2020; Rubin et al., 2020). In particular, there is a dearth of evidence regarding the risk of worsening of respiratory infection in patients receiving thiopurines. However, administration of thiopurine drugs is associated with an increased risk of opportunistic viral infections (Seksik et al., 2009; Harbord et al., 2017; Kirchgesner et al., 2018). Mercaptopurine has been shown to inhibit a protease that is essential for the maturation of Middle Eastern Respiratory Syndrome Coronavirus. However, there are no animal-based models that suggest the clinical efficacy of mercaptopurine (Cheng et al., 2015). Based on this finding, many studies have suggested that it is not necessary to discontinue thiopurines in stable patients (Dotan et al., 2020; Kennedy et al., 2020; Rubin et al., 2020).

Currently, there is no evidence based on the ethnicity, age, gender, or treatment to indicate the risks of exacerbation and mortality rates in patients with IBD who have contracted COVID-19. The Surveillance Epidemiology of Coronavirus Under Research Exclusion (SECURE-IBD) is an international database for monitoring and reporting the outcomes of COVID-19 in patients with IBD. With the aim of addressing the aforementioned issues, data on cases of IBD patients with COVID-19 are being accumulated in SECURE-IBD for use in various epidemiological studies and therapeutic investigations. Preliminary analyses based on the data obtained from SECURE-IBD have led to recommendations that the administration of immunosuppressants be refrained if a patient with IBD who is receiving immunosuppressant therapy, including thiopurines, contracts COVID-19. The treatment should be ceased until the patient recovers from the infection, owing to the high risk of worsened infections (Allez et al., 2020; Brenner et al., 2020). However, as the background of the patients was not examined in detail in these studies, further investigations are necessary in this regard.

## Conclusion

In this review, we described the effectiveness, side effects, and challenges of thiopurines in IBD. This review also discussed the use of thiopurine drugs during the COVID-19 pandemic. Thiopurines have been used for many years; however, with the emergence of anti-cytokine therapy in the IBD field, the importance of thiopurines has been revisited. However, there are no practical guidelines for clinicians regarding the use of thiopurines. An increasing number of analyses on TPMT and NUDT15 have elucidated the pharmacological diversity of drugs for which the metabolism is altered by the genetic makeup of an individual. The use of thiopurines as a key anchor drug for the treatment of IBD is expected to continue. Therefore, future research is necessary for providing evidence regarding the benefit–risk balance in cases where thiopurines are used in combination with a new drug and where the use of thiopurines is withdrawn from patients who have been receiving the drugs for a long period. The formulation of practical guidelines at a global level, by considering the ethnic and genetic background, is necessary for improved personalized medicine. It is also essential to establish evidence regarding the risk of infection, risk of exacerbation, suitability of treatment continuation, and drug interactions with concomitant medications, which would serve as a reference during disease pandemics, including COVID-19.

## Author Contributions

Conceptualization: KT. Writing the original draft preparation: KT. Writing review and editing: KT, TS, TT, and MK Supervision: MI and AI.

## Conflict of Interest

The authors declare that the research was conducted in the absence of any commercial or financial relationships that could be construed as a potential conflict of interest.
